# Imaging spectrum of lung adenocarcinoma with histopathological correlation

**DOI:** 10.1186/1470-7330-15-S1-P24

**Published:** 2015-10-02

**Authors:** V Noble, A Shah, F Mcleod

**Affiliations:** 1 Oxford University Hospitals NHS Trust, Windmill Road, Oxford, OX3 7LD, UK

## Learning objectives

The aim of this pictorial review is demonstrate the radiological appearances of adenocarcinoma with particular focus on more unusual appearances such as cystic adenocarcinoma.

## Content organisation

Non-small cell lung cancers account for almost 85% of all lung cancers and of these, adenocarcinoma is the most common. This entity has recently been reclassified to reflect increased understanding of the underlying pathology and thus it is crucial for radiologists to understand the new classification, the role of radiology in identifying pre-invasive lesions and the guidelines for management of subsolid nodules. We present the spectrum of imaging appearances from ground glass nodules (GGNs) to solid mass lesions with histopathological correlation.

## Conclusion

It is important for radiologists to recognise the spectrum of appearances of lung adenocarcinoma and follow appropriate algorithms for surveillance or further management.

